# When the Clinical Environment Is Closed to Students: The Harsh Reality of COVID-19 and Implications for Colleges of Osteopathic Medicine

**DOI:** 10.7759/cureus.12044

**Published:** 2020-12-12

**Authors:** Ken Heiles, Valerie Sheridan, Tami Hendriksz, Rebecca Giusti, Tyler C Cymet

**Affiliations:** 1 Family Medicine, Kansas City University of Medicine and Biosciences, Kansas City, USA; 2 Surgery, A.T. Still University, Mesa, USA; 3 Medical Education, Touro College of Osteopathic Medicine, Vallejo, USA; 4 Osteopathic Manipulative Medicine, Western University of Health Sciences, Pomona, USA; 5 Medical Education, American Association of Colleges of Osteopathic Medicine, Bethesda, USA

**Keywords:** medical education med ed learning classroom integrated, curriculum planning, covid 19, ward rotations, curriculum assessment, in-person rotations

## Abstract

When the World Health Organization (WHO) declared coronavirus disease 2019 (COVID-19) a global health emergency, Colleges of Osteopathic Medicine (COMs) debated the role of medical students during this developing pandemic. Initially, the discussion included whether medical students were essential personnel contributing to meaningful patient care. Many questions arose regarding how COVID-19 would affect medical education and if the changes would be temporary or continue for a significant period of time.

Due to the lack of availability of personal protective equipment (PPE) and a decreased focus on clinical education within many healthcare settings, in March the American Association of Colleges of Osteopathic Medicine (AACOM) declared that medical students were not essential personnel and recommended that COMs take a ‘pause’ and remove students from the clinical environment. This ‘pause’ would allow COMs time to assess where medical education could continue, to define the critical pieces of clinical education that required a clinical environment and to address how medical students could contribute during the pandemic.

The AACOM Clinical Educators group began to meet on a weekly basis during this time so that Clinical Deans from Osteopathic medical schools across the country could collaborate, share ideas, discuss current challenges, and co-create a system to deliver medical education realizing the limitations of in-person clinical training.

## Introduction

During these unprecedented times of severe disruption in osteopathic medical education, the American Association of Colleges of Osteopathic Medicine (AACOM) responded to the coronavirus disease 2019 (COVID-19) crisis by empowering new adaptive working groups of clinical leaders to address issues arising from the challenges in medical education resulting from the pandemic.

Three working groups addressing clinical education alternatives were formed: 1. Clinical Education Alternatives for Core Clinical Training, 2. Clinical Education Alternatives for Elective Rotations, and 3. Innovations and Transformations to Medical Education. The Educational Council on Osteopathic Principles (ECOP) also participated in the process to address issues of teaching osteopathic manipulative medicine within the restrictions required to teach responsibly during the pandemic. This paper reviews the common principles from and conclusions of the reports from the working groups.

## Technical report

Identifying issues

The COVID-19 pandemic brought a closure of clinical training sites abruptly ceasing in-person clinical education opportunities [1,2]. Medical educators were presented the opportunity to restructure and implement innovative approaches to deliver medical education content amidst challenging circumstances. The change occurring was similar to the move to the competencies and a shift from quantifying the time in education to focusing on the outcomes desired [3]. Requirements remained for students to complete the academic year and progress to the next level in their medical educational training [4].

This pause created an opportunity to consider the areas in need of close attention by osteopathic medical schools, and those that need to be outsourced to clinical facilities with varying connections to the colleges [5].

Required Core clinical experiences remain the foundation of the third year of medical school. These Core experiences may differ for each COM depending on their mission. Historically, Core clinical experiences occur in clinical environments where live healthcare is delivered. The most important issue for our osteopathic colleges centered on the ability to provide a high quality clinical educational experience in an environment where live, in-person interactions were no longer available. Simulation, standardized patient examinations and technology-based alternatives have been previously used as an adjunct to clinical experiences; however, they have never replaced the clinical experience. Specific healthcare interventions require touch and in-person interactions (Osteopathic Manipulative Medicine, incentive spirometry, intravenous catheter placement, arterial lines). Following purposeful discussions there was agreement within the Workgroup that a significant amount of medical knowledge content in each Core area of study could be learned outside of a clinical care facility.

Meeting the challenge of delivering and monitoring a high-quality educational experience, Core and Elective experiences were created in a distance learning fashion for students to continue their medical educational journey.

Core clinical clerkships in each of the clinical sciences are considered the foundation of a medical education. They include: Family Medicine, Internal Medicine, Surgery, Pediatrics, Obstetrics/Gynecology, Psychiatry, and Emergency Medicine. Elective clinical clerkships include medical and surgical subspecialties, radiology, pathology, research, and other fields that students are exposed to during their core clerkships.

Certain elective experiences: research, learning about patient safety and healthcare quality improvement issues, by their very nature do not need do not necessitate a live patient encounter. Third- and fourth-year electives typically serve as an opportunity for students to determine their interest as a future career in various specialty disciplines, as well as allowing students the opportunity for additional training and learning in areas best suited for their personal and professional goals. Distance learning electives were limited with little time to create them in a meaningful way. Many Colleges of Osteopathic Medicine (COMs) shared resources to provide the best standard curriculum to the students.

Fourth-year medical students are eager to identify opportunities to showcase their skills with residency programs where they are interested in matching. Most medical schools have limited connection with students in the fourth year from an educational perspective, so there may be missed opportunities for further education or identifying and filling knowledge gaps. Many COMs have limited control over the curriculum delivered in the fourth year during audition rotations, as some students appear to have diminished interest in training outside of rotations in their chosen specialty of interest. Educational assessment of the students can be challenging as each experience may vary widely in terms of quality or opportunity. Often, each COM has a lack of specialty chairs or dedicated faculty monitoring specific learning events and outcomes from sub-specialty rotations. During the pandemic, these varied opportunities were limited with COMs focusing on the requirements for progression and graduation. Since fourth-year students recently matched, the new focus was preparing the fourth-year students for graduation.

In preparation for the new academic year, more challenges have surfaced. Prometric testing sites have closed which resulted in fluctuations in availability of national board exam dates for students. Preparing for residency has now become even more complicated with the current national recommendations given by the Coalition for Physician Accountability Working Group on Student Movement [6]. These guidelines state that students should limit “away” rotations as much as possible for the 2020-2021 academic year. This requires leveraging the ‘in-network’ rotation and residency sites for the students to complete their training requirements for progression and graduation as well as matching into their desired residency programs. This issue has an especially negative impact on many osteopathic medical students such as military students, students with sub-specialty choices, and students within a distributed model of rural geographical locations as rotation schedules and graduate medical education (GME) opportunities are significantly less than for students in other geographic areas.

Most importantly, through these challenges, it is the duty of the COM to monitor the students to assess acquisition of a core body of knowledge, development of skills critical to practicing medicine, and achieving an understanding of the role of a physician as part of one’s professional identity formation combined with the use of the information technology.

How much patient interaction is enough?

Historically, on-site clinical rotations had significant variability in the depth and breadth of patient experience. Nothing replaces the in-person patient interaction for educational purposes to ensure that students are competent in all domains of patient interaction, interpersonal communication, professionalism, and performance of the physical and structural exam and certain clinical procedures as well as the competent execution of osteopathic manipulative treatment (OMT). Healthcare needs have changed, and people want to leverage technology in receiving healthcare. Incorporating this into training can be added, replace, or taught alongside education in a clinical setting [7].

Osteopathic physicians have a distinct approach to the patient that incorporates the osteopathic philosophy and principles, including the diagnosis and treatment of somatic dysfunction with OMT. The Educational Council of Osteopathic Principles (ECOP) stresses equal importance of cognitive knowledge foundation as well as the refinement and application of the psychomotor skillset. The ECOP recommends the following:

1. The didactic or cognitive component of the third- and fourth-year Osteopathic Principles and Practice (OPP) educational process may be met through a distance learning fashion.

2.This may include videos, manual or textbook readings, pre-recorded or interactive live-streaming lectures, and/or online modules at the discretion and resources of each COM.

3. The psychomotor skills component must be completed live and occur in person, at the discretion and resources of each COM. The ECOP recommends that the flexibility within the curriculum be promoted at this time of the COVID-19 pandemic so that once students are allowed to return to campus or the clinical learning environment, the COMs can provide make-up sessions for this psychomotor skills component of OPP.

Clinical rotations in other specialty disciplines could follow a similar approach with the blended on-site clinical experiences utilizing didactic opportunities, virtual patients, live interactive opportunities with live patients in a virtual Objective Structured Clinical Examination (OSCE) or telemedicine format, online learning modules, and simulation. The focus would be to offer a more in-depth distance learning curriculum on content and knowledge and offer application of the knowledge in the clinical settings. This approach allows the student an opportunity to gain a meaningful depth and breadth of the expected medical knowledge through the distance learning didactic experience. The didactic experience can be followed by the live patient interactions within the clinical learning environment occurring in an asynchronous manner later allowing fulfillment of academic requirements and achievement of all clinical learning objectives and application skills. The Working Group agrees that there does not seem to be a specifically defined amount of time that a student needs to spend in the clinical environment. However, it appears that there are specific skills that should be practiced and assessed in the clinical environment. Progressing into the 2020-2021 academic year, opportunities within the clinical learning environment will be ideal for the more prepared student to demonstrate their ability to interact in an appropriate manner in patient encounters and perform hands-on skills such as physical exam maneuvers and procedures (OMT, suturing, delivering a baby, venipuncture, etc.). Depending on the circumstances, the clinical experience may occur with simulated patients, telemedicine visits with actual patients or standardized patients, or other methods of training for human-like interaction all of which need to allow for outcome assessments and evaluation of competencies.

Solutions recommended by AACOM's Clinical Education Transformation Committees

For the Short-term Academic Year 2020-2021 Recommended Solution for Third-Year Medical Students.

Training

1. Students may have a blended didactic/clinical training experience in seven Core disciplines: Osteopathic Manipulative Medicine (OMM), Family Medicine (FM), Internal Medicine (IM), Pediatrics, Surgery, Obstetrics/Gynecology/Women’s Health, and Psychiatry. Note Emergency Medicine not included as a core clerkship.

2. Students could complete one to two weeks in a distance learning didactic experience followed by a two- to three-week on-site clinical experience totaling a four-week experience. Students would have additional didactic responsibilities assigned during the on-site clinical experience.

3. Students would take a subject matter examination like the Comprehensive Osteopathic Medical Achievement Test (COMAT) after completion of the distance learning and on-site clinical experience.

4. Longitudinal coursework may be necessary to fill gaps of knowledge or meet mission curricular requirements.

5. Elective rotations may be permitted in two-week time periods. This would allow more flexibility of scheduling and more subspecialty exposure for the students.

6. Students may be required to complete additional OSCE’s to allow additional evaluation of core competencies throughout the year.

7. A core curricular inventory designed by a consensus of the COMs being created as the second part of the clinical education transformation committee will identify ‘core patient cases and application skills’ that should be completed by every osteopathic medical student. Student patient logs should be maintained and would allow tracking of exposure to these key diagnoses/procedural skills. Students that lack direct patient interaction for any of the identified core cases or procedures listed would be expected to complete remediation later to fill these gaps which may include completing educational modules or related experiences to those diagnoses or procedures.

Blended training

As students transition back to onsite clinical experiences, it is expected that the volume of patients, and the depth and breadth of patient cases will likely be diminished. Virtual curriculum could be combined with the clinical experience to assure student exposure to “core” cases and procedures to fill expected gaps in medical knowledge.

For the Short-term Academic Year 2020-2021 Recommended Solution for Fourth-Year Students

1. Asynchronous training or blended training as described above for the third-year students may occur for the Core graduation requirements. These are designated at each COM.

2. Any number of audition rotations with parallel plans may be completed locally within the student’s geographical home base that are “in network” outside of VSAS/VSLO. VSAS/VSLO rotation approvals will be based on the national recommendations, at the site’s discretion.

3. Split rotations may be utilized for greater flexibility in scheduling and availability of opportunities.

4. Any recommended longitudinal coursework would continue based on each individual COM requirements.

5. A core curricular inventory designed by a consensus of the COMs will identify ‘core patient cases and application skills’ that should be completed by every osteopathic medical student. Student patient logs should be maintained and would allow tracking of exposure to these key diagnoses/procedural skills. Students that lack direct patient interaction for any of the identified core cases/procedures listed would be expected to complete remediation later to fill these gaps which may include completing educational modules or related experiences to those diagnoses/procedures.

6. Residency boot camps may be created and utilized for assessment of competencies and residency preparation purposes.

7. Development of milestone assessments based on the established EPA’s or AOA Core Competencies may be achieved in the fourth year to demonstrate competence, assist in student gap learning and validate preparation for residency.

Long-term academic year 2020-2021

Any existing longitudinal curriculum would proceed as outlined above.

Additional Objectives and Benefits to consider:

1. There is a need for common core knowledge to be identified and offered on more than the core clerkship training in order to prepare students for residency.

2. Students should be taught to Integrate content throughout in person and remote educational experiences such as oral communication skills, body language, and motivational interviewing skills for third- and fourth-year students.

3. Colleges of Osteopathic Medicine should move towards alignment with competency-based approaches to medical education.

4. A need to supplement the didactic experiences with Telemedicine experiences has been noted.

5. The expectation is that the ability to fully identify standards and have a greater degree of control for assessment purposes over the curriculum in the third and fourth years of the student's medical education.

6. Students will need to have the ability to build an internal library of experiences.

7. Students will need to replace lost clinical time for a student in their educational journey with other modes of education.

8. COM's and students should work to integrate ethics, professionalism, and other aspects that are the essence of becoming a physician.

9. COM's will need to work on the ability to effectively assess milestones, integrate curricular content not covered in other areas, and provide opportunity for inter-professional experiences, opportunities to better prepare the students for residency.

## Discussion

A successful medical education can be achieved in a variety of ways [8]. There was general agreement within the Working Group that a medical education that is delivered completely in a distance learning fashion would not be the same quality as a medical education that provides experience and exposure in the setting where care will eventually be delivered by the student trainee. There was also agreement that four-week clinical clerkships were one of many ways to achieve this goal. Historically, clinical experiences have occurred in a time-based manner. Rotations have been completed in a variety of weekly blocks allowing the student opportunities during that block of time. Clinical experiences can also be broken down into specific discrete objectives required to master a given clinical science or set of skills.

Throughout this pandemic, new challenges may present themselves and the osteopathic profession is prepared to maintain and deliver a high-quality education for our students. Regardless of any extenuating circumstances, it is important for each COM to determine a curricular inventory using the core curricular inventory designed by a consensus of the COMs that meets their mission and best prepares and monitors an osteopathic medical student to become a competent osteopathic physician. This may necessitate following each learner in a standardized way longitudinally throughout their medical education to identify any gaps in their medical knowledge and clinical skills. Student evaluations, despite the wide variability in the quality, depth, and attention paid to assessment by the clinical preceptors, remains a critical component to the assessment of the learner. If less time is spent in the clinical environment, it is up to each COM to assess their students by other means.

The Working Group proposes, that for each core area of study, there should be the creation of some system of content management and a system of assessment monitoring each content area. This may need to be done more frequently and in collaboration between faculty and clinical preceptors and performed longitudinally throughout the learner’s medical education journey to determine any gaps in a learner’s knowledge or performance. This may require another level of assessment in the student’s final year to ensure that medical knowledge and clinical skills do not remain compartmentalized and the student is able to utilize the skills attained.

Professionalism and teamwork were noted of utmost importance in all core areas of study. A variety of ways can be used to assess each learner for these important attributes; however, an assessment must include a certain amount of direct observation, simulated experiences or other assessment tools to link these key components of the core clinical sciences. The Working Group places this critical assessment of extreme importance for the learner to identify and understand their role as a future physician and in the formation of their professional identity.

## Conclusions

Medical Education requires didactic and clinical knowledge, application skills and professional identity formation. It has been determined that to achieve these three foundational pillars of a medical education, a combination of didactics and clinical in person encounters must occur. Online education is effective in the transmission of knowledge but is not adequate training by itself in the process of becoming a competent physician. Learning within each of the modified educational experiences, knowledge must be blended with exposure to healthcare in person where it is can be provided.

In order for all osteopathic medical students to achieve a similar medical education and training, efforts will continue to develop a core curricular inventory designed by a consensus of the COMS that will identify ‘core patient case diagnoses and application skills’ that should be completed by every osteopathic medical student ensuring a solid foundation of medical education.
